# 18F‐fluoro‐2‐deoxy‐2‐d‐glucose PET‐CT (FDG PET‐CT) in staging of high‐risk renal and urothelial bladder cancers (COPPER‐T) trial protocol

**DOI:** 10.1002/bco2.246

**Published:** 2023-05-10

**Authors:** Rahul Jena, Priyank Bhargava, Shashank Tripathi, Sameer Taywade, Taruna Yadav, Arjun Singh Sandhu, Mahendra Singh, Shiv Charan Navriya, Deepak Prakash Bhirud, Amit Aggarwal, Gautam Ram Choudhary

**Affiliations:** ^1^ Department of Urology All India Institute of Medical Sciences Jodhpur India; ^2^ Department of Nuclear Medicine All India Institute of Medical Sciences Jodhpur India; ^3^ Department of Diagnostic and Interventional Radiology All India Institute of Medical Sciences Jodhpur India

**Keywords:** bladder cancer, conventional imaging, FDG PET‐CT, high risk urothelial cancer, randomized trial, renal cell carcinoma

## Abstract

**Background and Study Design:**

Role of ^18^F‐fluoro‐2‐deoxy‐2‐d‐glucose positron emission tomography‐computed tomography (FDG PET‐CT) in evaluation of renal cell cancers (RCC) and urinary bladder cancers is not standardized, and the COPPER‐T trial, which is a single centre prospective randomized study, was designed to compare it with conventional imaging for staging of clinically localized high risk RCC and urinary bladder carcinoma (Stage T2 and above).

**Patients and Methods:**

There will be two subgroups of patients: RCC and urinary bladder carcinoma. In each of these, the patients will be randomized to either Arm A or Arm B. In each of the arms, each patient will be subjected to diagnostic imaging by FDG PET‐CT. The CT scan will be a contrast‐enhanced scan like that in conventional staging. A radiologist and nuclear medicine specialist will report the scan independently. The radiologist will not have access to the PET scan sequences and will only review the contrast‐enhanced computed tomography (CECT) images. In Arm A, the report of the conventional imaging modality, that is, CECT and bone scan if done, will be reviewed first by the clinician, and based on this report, a management plan will be made. Then, the PET‐CT report will be reviewed, and change in the management plan will be noted. New findings or equivocal findings if any in the PET‐CT report would be noted. In Arm B, the report of the PET‐CT report will be reviewed first by the clinicians, and a management plan will be made. Then, the CECT and/or bone scan reports will be reviewed, and any change in the management plan will be noted.

**Outcome and Significance:**

Final analysis of the data after completion of the trial will help in clarifying the role of FDG PET‐CT in high risk RCC and transitional cell carcinoma (TCC) of the bladder, its diagnostic accuracy compared with conventional imaging and the impact of using it on patient management.

## 1 INTRODUCTION

One third of all cases of renal cell carcinoma (RCC) have synchronous metastases at presentation.^1^ An additional 20–40% of patients with clinically localized disease at presentation will eventually progress and develop metastases.^2^ Metastatic RCC is almost always fatal with 5‐year survival rates of less than 15%.^3^ Therefore, accurate staging of newly diagnosed RCC is of paramount importance. Transitional cell carcinoma of the urinary bladder is the 12th most common malignancy.^4^ About 5–10% of all cases are metastatic at presentation.^5^ Almost 25–30% of all urinary bladder malignancies present with muscle invasive disease,^5^ and even with multidisciplinary decision‐making, there is a significant risk of under staging. Of the patients who undergo curative surgical resection, a significant proportion of patients ultimately experience disease relapse and progression with poor outcomes. This progression can be attributed to systemic disease at presentation which has escaped detection by conventional and recommended imaging modalities.

At present, both the National Comprehensive Cancer Network and European Association of Urology guidelines recommend contrast‐enhanced computed tomography (CECT) of the chest and abdomen for the initial staging of high risk RCC and urinary bladder cancer.^6^, ^7^, ^8^, ^9^ Bone scan is recommended only when there are symptoms suggestive of bony involvement or elevated alkaline phosphatase levels. Although ^18^F‐fluoro‐2‐deoxy‐2‐d‐glucose positron emission tomography (FDG PET) scan is an integral part of the staging and evaluation of many other cancers, its exact role in the evaluation and management of newly diagnosed RCC and urinary bladder cancer remains to be elucidated.^10^, ^11^ At present, both the National Comprehensive Cancer Network and European Association of Urology do not recommend the use of FDG PET as an initial staging modality in RCC and bladder cancer. Newer tracers, including those targeting fibroblast activation protein, are under evaluation in imaging of multiple cancers, including renal and urinary bladder cancers, and may prove to have higher sensitivity than FDG, but that is yet to be elucidated.^12^, ^13^ Nevertheless, there is a large body of literature in recent times that point towards the increased sensitivity of FDG PET‐CT in detecting distant metastases, compared with conventional imaging, in the initial staging of RCC and carcinoma urinary bladder, although this has not been proven in a head‐to‐head randomized controlled trial.^11^

## 2 COPPER‐T—CLINICAL TRIAL OVERVIEW

COPPER‐T, that is, COmParison of FDG PET‐CT with conventional imaging in the staging of high‐risk Renal cancers and Transitional cell carcinoma of bladder, is a single centre prospective randomized controlled trial that compares FDG PET‐CT to conventional imaging (CECT scan of the abdomen and bone scan wherever appropriate) for staging of clinically localized high risk RCC (>Stage T2) and urinary bladder malignancy (>Stage T2 and higher). The goal of this trial is to provide robust high‐level data on whether FDG PET‐CT should replace conventional imaging in the assessment of such patients or whether it should be used only in providing incremental diagnostic information in select cases. The hypotheses of this study are (a) FDG PET‐CT has improved diagnostic accuracy compared with conventional imaging modalities, (b) FDG PET‐CT should be used as the first line imaging modality in high risk RCC and bladder cancer and (c) the improved diagnostic accuracy of FDG PET‐CT will result in significant management impact in these patients. The primary objective of this study is to compare diagnostic accuracy of FDG PET‐CT to that of conventional imaging for detecting distant and nodal metastases in these patients. The secondary objectives have been listed below:

1. To compare the first line management impact of FDG PET‐CT to conventional imaging.
2. To compare the number of equivocal study results between the two modalities.
3. To assess the incremental accuracy of FDG PET‐CT or conventional imaging as a second line imaging modality by their ability to detect additional metastases over the first lime imaging performed.
4. To determine the impact of FDG PET‐CT or conventional imaging as a second line imaging modality over the first line imaging on the management of patients.

The tertiary objectives are the following:

1. To evaluate the prognostic value of FDG PET‐CT with regard to the disease‐free status (3 years from accrual of the last patient).
2. To report the acute adverse events for FDG PET‐CT if any.
3. To evaluate the utility of SUVmax in predicting histopathology and grade of RCC in the subset of patients with RCC.

## 3 PATIENTS AND METHODS

We aim to evaluate the role of FDG PET‐CT in patients of RCC and urinary bladder carcinoma with a significant risk of metastatic disease. Patients of RCC and urinary bladder carcinoma will be kept in different subgroups and will undergo separate analysis and follow‐up. The inclusion and exclusion criteria have been outlined below.

Inclusion criteria are as follows:

1. All patients attending the urology OPD of AIIMS, Jodhpur, and more than 18 years of age with imaging or biopsy findings suggestive of primary renal malignancy TNM Stage T2 and/or N1 and above.
2. All patients attending the urology OPD of AIIMS, Jodhpur, and more than 18 years of age with imaging or biopsy findings suggestive of muscle invasive carcinoma urinary bladder or N1 and above.
3. All patients giving written informed consent.
4. All patients willing and able to comply with the required procedures of the study in the opinion of the investigator.

Exclusion criteria are as follows:

1. Any patient not giving consent for the study.
2. Any patient with poor performance status or life expectancy excluding him/her from curative surgery or radiotherapy or use of oral/intravenous therapy.
3. Known case of renal, upper tract urothelial cancer (UTUC) or bladder malignancy post curative intent.
4. Known case of metastatic malignancy already on treatment for advanced disease.
5. Patient with compromised renal function in whom administration of intravenous (IV) iodinated contrast is contraindicated.
6. Patient with known hypersensitivity to the radiotracer or contrast agent to be used.
7. A history of other known malignancy within the last 2 years with the exception of melanoma or any other known skin cancer.

The study is approved by the All India Institute of Medical Sciences, Jodhpur Ethics Committee and is prospectively registered in the Clinical Trials Registry of India and WHO ICTRP bearing trial no. CTRI/2022/07/043691.

## 4 PROCEDURE

There will be two subgroups of patients: RCC and urinary bladder carcinoma. In each of these subgroups, the patients will be randomized to either Arm A or Arm B by computer‐generated randomization. In each of the arms, each patient will be subjected to diagnostic imaging by FDG PET‐CT. Patients with elevated alkaline phosphatase or symptoms suggestive of bony involvement will also undergo a bone scan within a week of the PET‐CT scan.

### 4.1 Acquisition and sequence of FDG PET‐CT scan

The patients will be kept nil per oral 8 h before the scheduled scan. If any patient will be diabetic, antidiabetic medications will be withheld only on the day of the scan. Blood glucose levels will be measured just before the scan and should be <200 mg/dL. Along with this, the patient's weight and height will be recorded followed by F‐18 FDG administration (0.150.2 mCi/kg body weight) through an 18‐gauge IV catheter. Patients will then be kept in the post injection room and will be ask to take adequate oral fluids for hydration for better background clearance and less radiation retention. Whole body PET‐CECT images will then be acquired from vertex to midthigh in a whole‐body 128 slice GE MIDR PET‐CT scanner, 60–90 min post injection of radiotracer. Scout tomogram will be taken followed by breath‐hold noncontrast scan of thorax, abdomen and pelvis. IV‐iodinated contrast (iohexol—*contrapaque‐300* or iodixanol—*cardiolek‐320*) will be given at the rate of 1.5–2 mL/kg (maximum ~150 mL). For renal mass evaluation, three‐phase images will be acquired in order as follows: cortico‐medullary phase of abdomen–pelvis (~35 s post injection), followed whole body CECT in nephrogenic phase (~70–75 s post injection), whole body PET (12 min/bed, 8–10 beds) and delayed CT of abdomen–pelvis (~15–20 min post injection). Post diuretic dual‐point PET/CT imaging of abdomen will be done ~45–60 min after the whole body PET/CT acquisition. For bladder mass evaluation, two phase image will be acquired as follows: whole body CECT images (venous phase ~60–65 s post injection), followed by whole PET acquisition (1–2 min/bed, 8–10 beds) and delayed CT of abdomen and pelvis (~15–20 min post injection). Interpretation of PET‐CT images will be done by visual and semiquantitative (SUVmax) analysis.

Images of all sequences of the FDG PET‐CT will then be provided in a compact disc (CD) drive for interpretation. A nuclear medicine specialist will review the FDG PET‐CT and provide a detailed written report as per established departmental format for reporting. A radiologist will review the images of the CECT sequences and will provide a detailed report as per established reporting format. Both the reports will be prepared independent of each other, that is, the radiologist will be blinded to the report given by the nuclear medicine specialist and vice versa. Both these reports will then be sequentially reviewed by two clinicians as per the schema described in the following paragraphs.

In Arm A, for each patient, the report of the conventional imaging modality, that is, CECT and bone scan if done, will be first reviewed by the clinicians, and based on this report, a management plan will be made. Then, the PET‐CT report will be reviewed, and change in the management plan if any will be noted. New findings or equivocal findings if any in the PET‐CT report would be noted. In Arm B, the report of the PET‐CT report will be reviewed first by the clinicians, and a management plan will be made. Then, the CECT report and the report of the bone scan if any will be reviewed, and any change in the management plan will be noted.

Positive scans for distant metastases or loco‐regional lymph nodes are to be identified using the following criteria (any one of the following):

1. Histopathology demonstrating metastatic malignancy.
2. Typical appearance of metastatic disease on scan.
3. Presence of lesion correlating with clinical signs and symptoms.

Confirmation of metastatic lesions demonstrated on scans is strongly encouraged using biopsy or other imaging modalities. However, this is not binding and is left to the discretion of the clinicians. Management impact will be classified as follows:

- High impact: Change in management intent or modality.
- Medium impact: Change in delivery of modality, but not intent.
- Low impact: Management plan was not altered.
- Potential impact ignored: Management plan was not altered despite positive findings in the second reviewed report after negative first report.

### 4.2 Follow‐up imaging

Follow‐up imaging for those that undergo curative treatment would be done for all patients using the same sequence of FDG PET‐CT at 6, 18, 30, 42 and 54 months. Initiation of any adjuvant or salvage treatment, appearance of local or distant metastatic disease or death of the patient or loss to follow‐up would also be noted.

### 4.3 Clinical management

Clinical management of patients within this study is at the discretion of two individual clinicians working within a multidisciplinary framework. The study protocol does not determine how patients are managed; however, the management impact of each imaging modality is recorded by the treating clinician after reviewing the reports of each scan. As these patients are being considered for localized treatment at the time of inclusion, it is expected that the majority of patients within the study will proceed to radical surgery. Pathological findings at surgery will be compared with imaging.

### 4.4 Sample size and power calculation for the primary end point

Sample size calculation was done for the primary outcome, that is, diagnostic accuracy for PET‐CT scan and conventional CT scan. We calculated the sample using below mentioned formula (effect size) δ = (area under the curve for PET‐CT scan) AUC_1_ − (area under the curve for conventional CT scan) AUC_2_ with an effect size (δ) of 0.15, 95% confidence interval and 80% power. For RCC, we assumed AUC_1_ to be 0.75, and a sample size of 134 was calculated. Considering total dropout rate of 10%, a final sample size of 148 was reached. Similarly, for bladder cancer, sample size of 174 were estimated using AUC_1_ of 0.70, an effect size (δ) of 0.15, 95% confidence interval and 80% power.

The primary and secondary end points are enumerated as follows:

- Accuracy: The diagnostic accuracy of combined FDG PET‐CT versus that of conventional imaging will be evaluated as the primary end point of the study. Positive scans will be identified using at least one of the criteria enumerated earlier. Accuracy will be assessed by the area under the receiver operator characteristic curve (the AUC). For the purpose of estimating AUC, specificity and sensitivity, all scans reported as equivocal will be considered as negative.
- Management impact: The clinicians will make a baseline plan after reviewing the reports of the conventional imaging and PET‐CT scan as per the sequence in each arm. After viewing the first report, a plan is made and any change in the management plan made after viewing the second report will be recorded, and the impact will be noted as described above. The proportion of patients requiring a change in management will be compared between the two arms using an exact test to compare two independent binary variables.
- Incremental accuracy of second line imaging: In our study, a single test will be performed in which FDG PET and CECT will be combined. Therefore, in each arm, second line imaging will be used to denote that test whose report will be reviewed second, and first line imaging will be the test whose report is reviewed first. In Arm A, conventional imaging, that is, CECT and bone scan if done, will be the first line imaging, and FDG PET‐CT will be the second line imaging. In Arm B, FDG PET‐CT will be the first line imaging, and CECT and bone scan if done will be second line imaging. The incremental accuracy of second line imaging will be its ability to uncover disease that was not discovered by the first line imaging. For this, a single radiologist would be reporting all the CECT scans, and a single nuclear medicine specialist would be reporting all the PET‐CT scans. For each arm, the proportion of all patients who were upstaged to M1 disease from M0 or all patients with M1 disease on first line imaging who had additional metastases detected on second line imaging will be estimated.
- Equivocal studies: Any scan in which the distant lesion or none of the lesions in case of multiple lesions has been reported to be equivocal for metastatic nature will be considered an equivocal study.
- Prognostic value: The prognostic value of FDG PET‐CT is the ability of a negative PET‐CT at baseline to predict the time till recurrence, that is, development of distant or local recurrence following definitive therapy. Kaplan–Meier curves will be plotted for the time until the appearance of distant or locally recurrent disease.
- Adverse events: Any adverse events recorded by the patient will be recorded, defined as those experienced by the patient at the time of radiotracer injection and during the 2 h following injection. The adverse events will be graded as per the NCI Common Terminology Criteria for Adverse Events v5.0 and its relationship to the diagnostic imaging will be described as unrelated, unlikely, possible, probable or definite. The worst grade of each toxicity for each patient within the toxicity assessment window will be tabulated.

## 5 DISCUSSION

Classically, RCC and urothelial carcinoma of the urinary bladder were not evaluated with FDG PET scans because activity of RCC with FDG is equivalent to or slightly higher than normal renal parenchyma, making detection difficult, and because of physiological excretion of FDG in pelvicalyceal system and urine, thus obscuring urothelial and renal cell cancers.^11^, ^14^ Another question which arises regarding use of FDG PET‐CT is its cost effectiveness and potential side effects related to radiotracer administration, although it is relatively safe. It has been postulated that use of PET‐CT is cost effective in the long run and helps in avoidance of unnecessary diagnostic and therapeutic interventions as a result of its anato‐metabolic role.^15^

Multiple recent studies have shown FDG PET‐CT to be at par, if not better in evaluation of primary and recurrent tumours with lymph node involvement, monitoring response to treatment and metastatic tumour burden as compared with CECT alone.^16^, ^17^ Impact on patient care and change in patient management with FDG PET‐CT as compared with conventional imaging has been investigated recently, especially for patients with urothelial carcinoma, and more than 70% of the patients with urothelial carcinoma of urinary bladder had a change in their management plan.^18^ Such numbers have guided researchers to move towards precision medicine in patients with muscle invasive bladder cancers using FDG PET‐CT with good results.^19^ The same significant impact on management of RCC is yet to be conclusively proven after inclusion of FDG PET‐CT as one of the imaging modalities in its evaluation.

Usual comparison between FDG PET‐CT and conventional imaging in patients with high risk RCC and TCC of the bladder would have required serial imaging leading to double radiation exposure, more use of manpower and resources and longer time. Considering the resources available and existing burden on the current health infrastructure in our country, we devised this protocol of combining FDG PET and CECT into a single time scan, which would solve our purpose utilizing the resources and personnel in the most effective manner, answer all our potential queries and simultaneously providing level 1 evidence clarifying the role of FDG PET‐CT in these patients. One of the tertiary objectives in this study is to assess for potential adverse effects of using such an imaging protocol, and final data from this study will also provide safety data for the same.

We are hopeful that final analysis of data after completion of the trial will help in clarifying the role of FDG PET‐CT in high risk RCC and TCC of the bladder and tailor medicine in these patients.

## AUTHOR CONTRIBUTIONS

*RJ, GRC, ST* conceived the study. *RJ, GRC, ST, ST, TY, PB* initiated the study design and *PB, SCN, MS, DPB, AA* and *ASS* helped with implementation. All authors contributed to refinement of the study protocol and approved the final manuscript.

## CONFLICT OF INTEREST STATEMENT

The authors have no conflicts of interests to declare.

## TRIAL REGISTRATION

The study is approved by Institute Ethics Committee and is prospectively registered in the Clinical Trials Registry of India and WHO ICTRP bearing trial no. CTRI/2022/07/043691.
